# Unifying AI-assisted scientific discovery around exploration, hypothesis generation, and testing

**DOI:** 10.1016/j.patter.2026.101660

**Published:** 2026-09-28

**Authors:** Md Musaddaqul Hasib, Sumin Jo, Harsh Sinha, Jifeng Song, Arun Das, Zhentao Liu, Hugh Galloway, Huey Huang, Jianqiu (Michelle) Zhang, Kexun Zhang, Shou-Jiang Gao, Yu-Chiao Chiu, Lei Li, Yufei Huang

**Affiliations:** 1 Cancer Virology Program, UPMC Hillman Cancer Center, Pittsburgh, PA, USA; 2 Department of Medicine, University of Pittsburgh, Pittsburgh, PA, USA; 3 Department of Electrical and Computer Engineering, Swanson School of Engineering, University of Pittsburgh, Pittsburgh, PA, USA; 4 Intelligent Systems Program, School of Computing and Information, University of Pittsburgh, Pittsburgh, PA, USA; 5 Electrical and Computer Engineering, University of Texas at Austin, Austin, TX, USA; 6 Language Technologies Institute, Carnegie Mellon University, Pittsburgh, PA, USA; 7 Department of Microbiology and Molecular Genetics, University of Pittsburgh School of Medicine, Pittsburgh, PA, USA; 8 Department of Computational and Systems Biology, University of Pittsburgh School of Medicine, Pittsburgh, PA, USA; 9 Cancer Therapeutics Program, UPMC Hillman Cancer Center, Pittsburgh, PA, USA; 10 Klesse College of Engineering and Integrated Design, University of Texas at San Antonio, San Antonio, TX, USA

**Keywords:** AI for scientific discovery, the EXHYTE framework, large language models, hypothesis generation, idea generation, AI for hypothesis testing

## Abstract

Large language models (LLMs) and agent systems increasingly support scientific discovery across domains. Yet the literature remains fragmented around isolated tasks, with limited attention to how components form integrated workflows. Here, we introduce exploration, hypothesis generation, and testing (EXHYTE) as an empirical workflow abstraction for reviewing AI-assisted scientific discovery. We mapped a corpus of recent studies to EXHYTE stages and substages to identify recurring strategies, workflow connections, and capability gaps. The analysis shows strong progress in exploration, especially retrieval, knowledge assembly, and representation learning as well as growing capacity for hypothesis and idea generation. The central bottleneck remains the transition from hypothesis or idea generation to executable experimental or computational testing, followed by feedback that can refine subsequent workflow steps. Because EXHYTE organizes systems by workflow function rather than agent architecture, it applies to both multi-agent scaffolds and emerging generalist tool-using agents. It also shows that integrated workflows do not imply full automation: current systems still depend on human judgment for problem formulation, contextualization, interpretation, risk assessment, and oversight. We discuss evaluation, reproducibility, hypothesis-space narrowing, external validation, and governance considerations.

## The bigger picture

Artificial intelligence is beginning to influence how scientists search for evidence, identify promising questions, generate explanations, and design experiments. However, progress in solving individual tasks does not necessarily translate into reliable scientific discovery. Scientific progress requires hypotheses to be translated into feasible test cases, evaluated against reliable evidence, and revised when results do not support them. Our survey shows that this connection remains the central weakness of current AI-assisted discovery systems. Retrieval, knowledge organization, and idea generation are advancing rapidly, but comparatively few systems can convert a promising hypothesis into an executable experimental or computational plan and then use the resulting evidence to guide the next step. By organizing the field around exploration, hypothesis generation, and testing, our process-centric perspective makes this bottleneck visible and shifts attention from isolated demonstrations toward complete, transparent, and auditable discovery workflows. Closing these gaps will require better experimental interfaces, stronger and extensive validation, reproducible outcomes, and reliable feedback mechanisms that allow AI systems and scientists to learn from unsuccessful as well as successful results.

## Teaser

In this review, Hasib et al. introduce EXHYTE a process centric survey to unify research on AI-assisted scientific discovery. Mapping recent studies reveals rapid advances in evidence retrieval, knowledge assembly, and idea generation, but it also reveals a persistent bottleneck in translating hypotheses into executable tests and using results to refine subsequent steps. The survey underscores the need for human oversight, reproducibility, validation, and auditable end-to-end discovery workflows.

## Introduction

Recent advances in large language models (LLMs) have accelerated applications in scientific discovery across chemistry,^1^^,^^2^ biology,^3^ materials science,^4^^,^^5^ and physics.^6^ Despite this progress, many systems remain organized around isolated tasks, such as literature retrieval, idea generation, experimental design, or automated testing. Existing surveys likewise often summarize methods by task or domain.^7^^,^^8^ Such views leave three questions unanswered: how individual AI methods are composed into broader discovery workflows, where capabilities concentrate, and where transitions between stages remain weak.

To address this gap, we introduce the exploration, hypothesis generation, and testing (EXHYTE) framework, an empirical, process-centric lens for reviewing AI-assisted scientific discovery (Figure 1). Rather than positing a universal scientific method^9^ or prescribing how inquiry should proceed, EXHYTE abstracts a recurring operational pattern from recent LLM- and agent-based systems. Across the surveyed literature, these systems commonly begin by exploring scientific knowledge or data through retrieval, structuring, and synthesis; proceed to generate candidate hypotheses, ideas, designs, or interventions; and then test or refine these candidates through computational, experimental, or expert-mediated evaluation. This pattern aligns broadly with accounts that emphasize the iterative, exploratory, and data-intensive character of scientific inquiry,^10^^,^^11^ but its purpose is narrower: to describe how current AI-for-science systems organize and connect discovery-related activities. EXHYTE formalizes the pattern as an iterative framework rather than a fixed sequence, and at substage granularity, it provides a common vocabulary for comparing methods, identifying where AI capabilities concentrate or remain underdeveloped, and analyzing how systems connect, or fail to connect, different parts of the discovery process.

**Figure 1:**
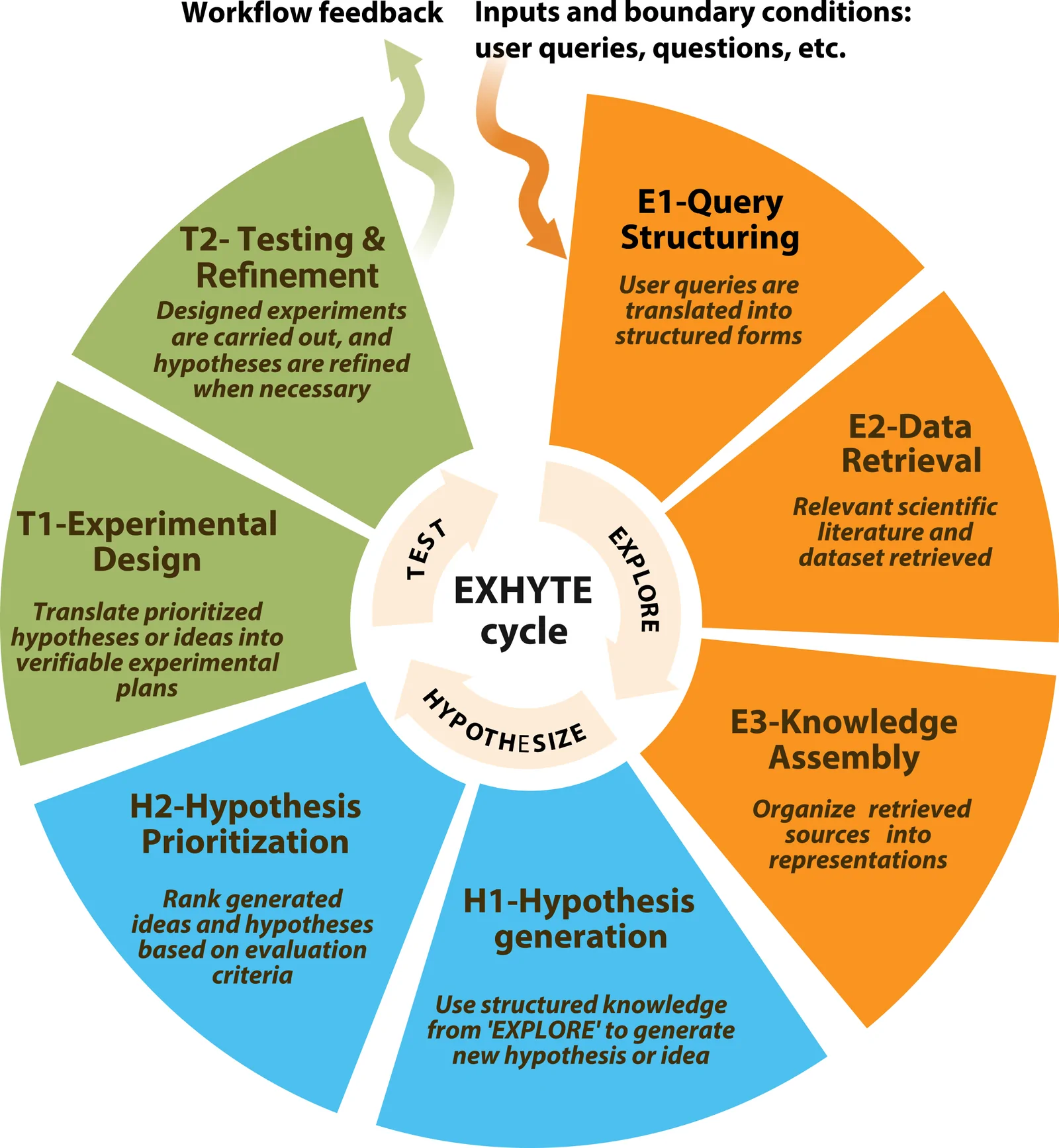
Overview of EXHYTE as an empirical workflow abstraction for AI-assisted scientific discovery Inputs and boundary conditions initiate or constrain the workflow. Outputs from testing can provide workflow feedback by updating the knowledge base, refining candidate hypotheses or designs, adjusting evaluation plans, or motivating additional exploration.

This workflow-level view also clarifies the boundary between integration and full automation. A system may connect multiple EXHYTE stages while still relying on human judgment for problem formulation, hypothesis expansion, interpretation of ambiguous evidence, and decisions about whether and how to revise hypotheses, adjust evaluation plans, or seek additional evidence. These dependencies are not incidental implementation details; they reveal where current systems automate technical subtasks while still requiring human-provided boundary conditions and checkpoints.

In this paper, we survey existing LLM- and agent-based methods through the lens of EXHYTE. We categorize the strategies proposed for each substage, examine how methods from different areas combine into human-in-the-loop or closed-loop systems, and identify workflow-level bottlenecks that limit reliable end-to-end discovery. By grounding the framework in empirical patterns from current AI-for-science practice, our survey clarifies how LLMs and agents contribute to scientific discovery and how these systems may advance toward more reliable, transparent, and adaptive workflows. An accompanying website at https://webapps.crc.pitt.edu/exhyte/ provides paper summaries and an EXHYTE-based interactive survey.

### The EXHYTE framework: An empirical workflow abstraction for AI-assisted scientific discovery

This survey uses EXHYTE as a workflow abstraction for organizing and comparing AI-for-science systems (Figure 1). We derived its stages through a comparative analysis of recent LLM- and agent-based research systems, in which a recurring operational structure appears across domains: scientific knowledge or data are first retrieved, organized, or analyzed; candidate hypotheses, ideas, designs, or interventions are then generated; and these candidates are subsequently evaluated through computational, experimental, or expert-mediated testing. EXHYTE formalizes this observed design pattern as a review framework for mapping system capabilities, not as a comprehensive account of scientific method.

This distinction matters. Scientific inquiry also involves problem formulation, theoretical framing, anomaly recognition, conceptual change, instrument development, social validation, and judgments about which questions are worth pursuing. Many of these activities are only partially represented in current AI-for-science systems, and several remain dependent on human researchers. EXHYTE should therefore be read as an empirical abstraction of the systems surveyed here, not as a claim that all discovery reduces to an explore-hypothesize-test sequence.

Research typically enters the EXHYTE workflow through an input or boundary condition: an open question, an unexpected observation, an anomaly in data, a new measurement technology, a dataset, a paper collection, or a user-specified research goal. We treat these inputs as boundary conditions because most existing systems still rely on human researchers to define the initial problem space, select contextual materials, specify constraints, and judge whether a question is worth pursuing. This boundary does not make problem formulation trivial; rather, it identifies automated support for problem formulation, anomaly recognition, and research-priority assessment as important open challenges for AI-assisted discovery.

Once an input has been specified, the first stage, EXPLORE, organizes what is known and exposes what remains uncertain. It assembles and structures knowledge so that gaps, unresolved questions, empirical patterns, or candidate search spaces become explicit and actionable. This stage consists of three substages.

• E1: Query structuring translates research questions, goals, or inputs into machine-actionable forms, such as key phrases, controlled vocabularies, domain schemas, or decomposed sub-queries, to guide retrieval and analysis.
• E2: Data retrieval collects literature, code, structured datasets, molecular or materials records, and other scientific resources from repositories, databases, and APIs according to the structured query plan from E1.
• E3: Knowledge assembly normalizes and integrates retrieved information into representations such as embeddings, graphs, feature tables, structured summaries, or curated databases, surfacing gaps, patterns, or candidate directions.

The output of EXPLORE is a structured knowledge base, set of retrieved resources, or empirical representation that supports downstream generation of candidate hypotheses, ideas, or designs.

HYPOTHESIZE transforms knowledge gaps, empirical patterns, or design opportunities into candidate ideas or specific claims. It includes two substages.

• H1: Hypothesis/idea generation formulates candidate mechanisms, explanations, designs, or interventions through approaches such as literature-grounded reasoning, multi-agent synthesis, knowledge-graph reasoning, or data-driven pattern discovery.
• H2: Hypothesis/idea prioritization evaluates these candidates for properties such as novelty, plausibility, feasibility, expected impact, testability, or alignment with domain constraints, using LLM-based evaluation, literature-based assessment, quantitative domain metrics, or expert review.

The outcome of HYPOTHESIZE is a ranked or filtered set of candidate hypotheses, ideas, or designs with rationales, evidence links, and, when available, success criteria for evaluation.

TEST translates selected candidates into executable evaluations and produces evidence that informs subsequent workflow steps. It includes two substages.

• T1: Experimental design specifies objectives, protocols, datasets, materials, computational workflows, and measurable outcomes, yielding executable experimental or computational plans.
• T2: Testing and refinement performs *in silico*, *in vitro*, *in vivo*, or expert-mediated evaluation and analyzes the results to assess, revise, or reject candidate hypotheses, ideas, or designs.

The output of TEST may include quantitative results, experimental observations, expert assessments, error analyses, updated evidence records, or revised candidate descriptions. In a workflow setting, these outputs can update the assembled knowledge base, refine candidate hypotheses or designs, adjust evaluation protocols, or identify additional information needed for subsequent analysis. Feedback in EXHYTE should thus be understood as a practical mechanism for linking evaluation results back to earlier workflow components, not as a philosophical claim that evidence mechanically confirms or falsifies hypotheses.

Taken together, the three stages provide a structured vocabulary for analyzing the current AI-for-science literature. EXHYTE lets us ask where existing systems concentrate their capabilities, which substages remain underdeveloped, how components connect across the workflow, and where human oversight remains necessary for problem choice, evidence interpretation, risk assessment, and scientific value judgment. In this sense, EXHYTE functions as both a review framework and a design map for AI-assisted discovery workflows.

### AI methods across the EXHYTE workflow

To construct the survey corpus, we focused on the literature published between 2018 and July 2025 to capture recent advances in LLMs and agent systems for scientific discovery. Articles were retrieved through keyword searches on arXiv, bioRxiv, and Semantic Scholar APIs, using queries such as LLM, idea generation, hypothesis generation, scientific discovery, experiment design, material discovery, molecular design, drug discovery, and biomedical discovery. Retrieved results were then manually filtered to remove irrelevant articles and ensure inclusion of high-quality papers from major conferences and journals. The final corpus comprises 87 papers spanning diverse disciplines. Because research in this area is advancing rapidly, a number of relevant papers released after our July 2025 cutoff could not be included in the analyzed corpus; to keep the survey current, these newer studies are incorporated into the accompanying website, which we update on an ongoing basis.

Because EXHYTE is used here as an empirical abstraction of observed AI-for-science workflows, our analysis proceeded in two steps. First, we mapped each paper to the broad workflow functions it addressed: EXPLORE, HYPOTHESIZE, and TEST. Second, we identified recurring implementation strategies within each function and grouped them into substages. This procedure allowed the framework to emerge from observed design patterns in the technical literature rather than from a presupposed general theory of scientific method.

The publication volume in this area surged between 2023 and 2024 and continued to rise sharply in 2025 (Figure 2A), reflecting both growing cross-domain interest and rapid methodological progress in applying AI to scientific discovery (Figure 2D). Across subject areas, most papers come from Applied Sciences and Engineering, followed by Social, Chemical, and Biological Sciences (Figure 2B). Based on our systematic review of the corpus, many papers progress beyond exploration to the HYPOTHESIZE stage, yet fewer continue to the TEST stage (Figure 2C). This disparity highlights a persistent disconnect between hypothesis or idea generation and empirical or computational evaluation in contemporary AI-assisted scientific discovery.

**Figure 2:**
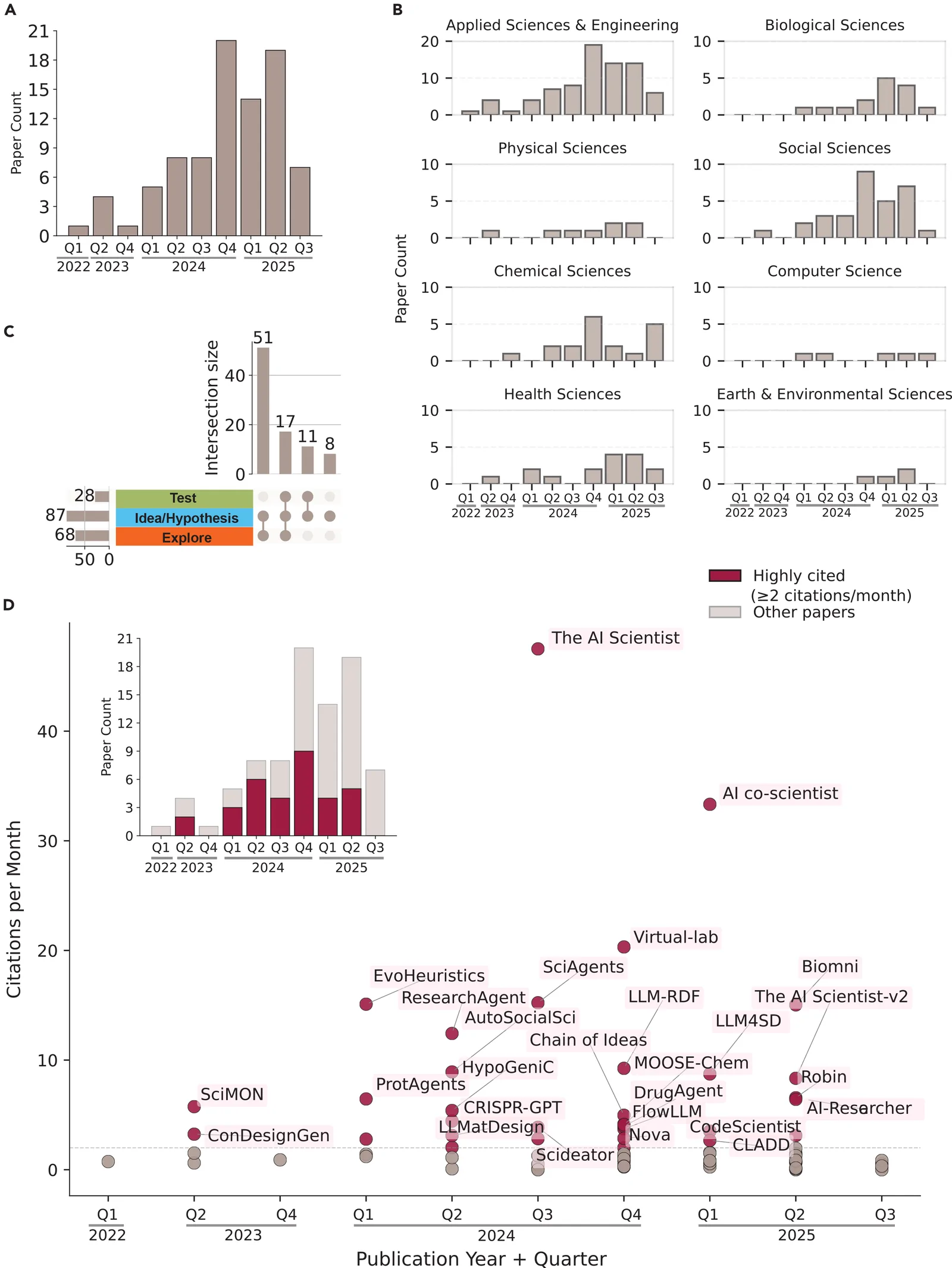
Temporal and thematic analysis of publications (A) Quarterly count of publications over time. The bar height represents the number of papers published in each quarter. (B) Quarterly distribution of publications by subject area. Bars indicate the number of papers in each subject area per quarter. (C) Upset plot showing the number of publications covering different stages of the EXHYTE framework (Explore, Idea/Hypothesis, Test). Bars represent the number of papers in each combination of stages. (D) Scatterplot of highly cited papers (red dots), with citations per month on the *y* axis and quarter/year on the *x* axis. Highly cited papers are defined as those receiving ≥2 citations per month. The red bars in the overlaid bar plot indicate the number of highly cited papers per quarter.

To systematically analyze and organize this rapidly expanding body of work, we identified recurring AI strategies that address challenges in each EXHYTE substage. Organizing the literature around these strategies moves beyond task- or domain-specific taxonomies and provides a structured view of how AI methods operate across the workflow. In the following sections, we present these strategies substage by substage, defining core patterns, summarizing representative studies and domain-specific variants, and highlighting how each approach is implemented and supported by empirical evidence.

#### Inputs and boundary conditions for EXHYTE workflows

In automated scientific discovery systems, inputs range from natural language queries and high-level research goals to domain-specific papers, datasets, structured specifications, and unexpected observations. In the EXHYTE framework, these inputs are treated as boundary conditions for the workflow rather than as evidence that the system has autonomously formulated a research problem. This distinction is important because many current systems still depend on human researchers to define the initial problem space, provide contextual materials, specify constraints, recognize anomalies, or determine which questions are worth pursuing. Thus, placing inputs at the boundary of EXHYTE is not meant to make problem formulation or anomaly recognition appear trivial. Rather, it makes visible a current limitation of AI-for-science systems: upstream problem framing and research-priority judgment remain only partially automated.

High-level research directions, such as workshop themes, set the scope for the automated scientific workflow. For example, the ICLR 2025 workshop “I Can’t Believe It’s Not Better” (ICBINB) focused on real-world pitfalls, challenges, and negative or inconclusive results in deep learning, a perspective incorporated in AI-Scientist-v2.^12^ Similarly, focused problem statements, such as “How can we improve the energy efficiency of AI models?” (e.g., SCI-IDEA^13^), guide idea and hypothesis generation. Extending this idea, IRIS^14^ formalizes user input as research goals that are explicitly transformed into a problem and its motivation/context, for example, the research goal “To improve the balance between quality and diversity in text generation (problem) by addressing the limitations of existing sampling methods like top-p (nucleus sampling), which often produce incoherent or repetitive outputs at higher temperatures (motivation/context).” Structured, domain-specific inputs similarly allow users to express goals and constraints in scientific formats. For example, LLMatDesign^5^ begins with user-specified chemical compositions and target properties for candidate materials, while AutoMAT^15^ accepts user-specified targets such as yield strength, density, or elemental cost constraints. In drug discovery, systems such as RAG-Enhanced Collaborative LLM Agents for Drug Discovery^16^ operate on query molecules represented as SMILES strings, and PiFlow^17^ integrates foundational scientific laws or expert-defined principles to guide search.

Beyond explicit goal specification, user-provided resources such as research papers and datasets function as empirical and contextual inputs. Systems like ResearchAgent,^18^ CodeScientist,^19^ and HypER^20^ leverage literature for retrieval and idea grounding. CodeScientist,^19^ for example, summarizes vetted code blocks to ensure that generated research ideas are practical, implementable, and executable. Similarly, domain-specific datasets provide an empirical foundation for discovery. DrugMCTS,^21^ for example, employs molecule-protein interaction datasets such as DrugBank^22^ and KIBA^23^ to support drug discovery tasks.

Together, these examples show that inputs are not merely technical prompts that initiate the EXHYTE workflow. They shape the scope, direction, and epistemic limits of downstream EXHYTE. When users heavily pre-load a system with selected papers, datasets, assumptions, or domain constraints, the resulting workflow may become less sensitive to alternative hypotheses, overlooked evidence, or competing empirical paradigms. For this reason, EXHYTE treats inputs as boundary conditions that reveal, rather than hide, the dependence of current AI-for-science systems on human problem formulation, contextualization, and judgment.

#### The Explore stage

##### E1: Query structuring

User query structuring is the first step in the Explore stage, where it transforms raw, unstructured research inputs into actionable representations. This process employs complementary strategies including decomposition, vector representation, and information extraction from datasets to enable targeted exploration across literature and data (Figures 3A and 4).

**Figure 3:**
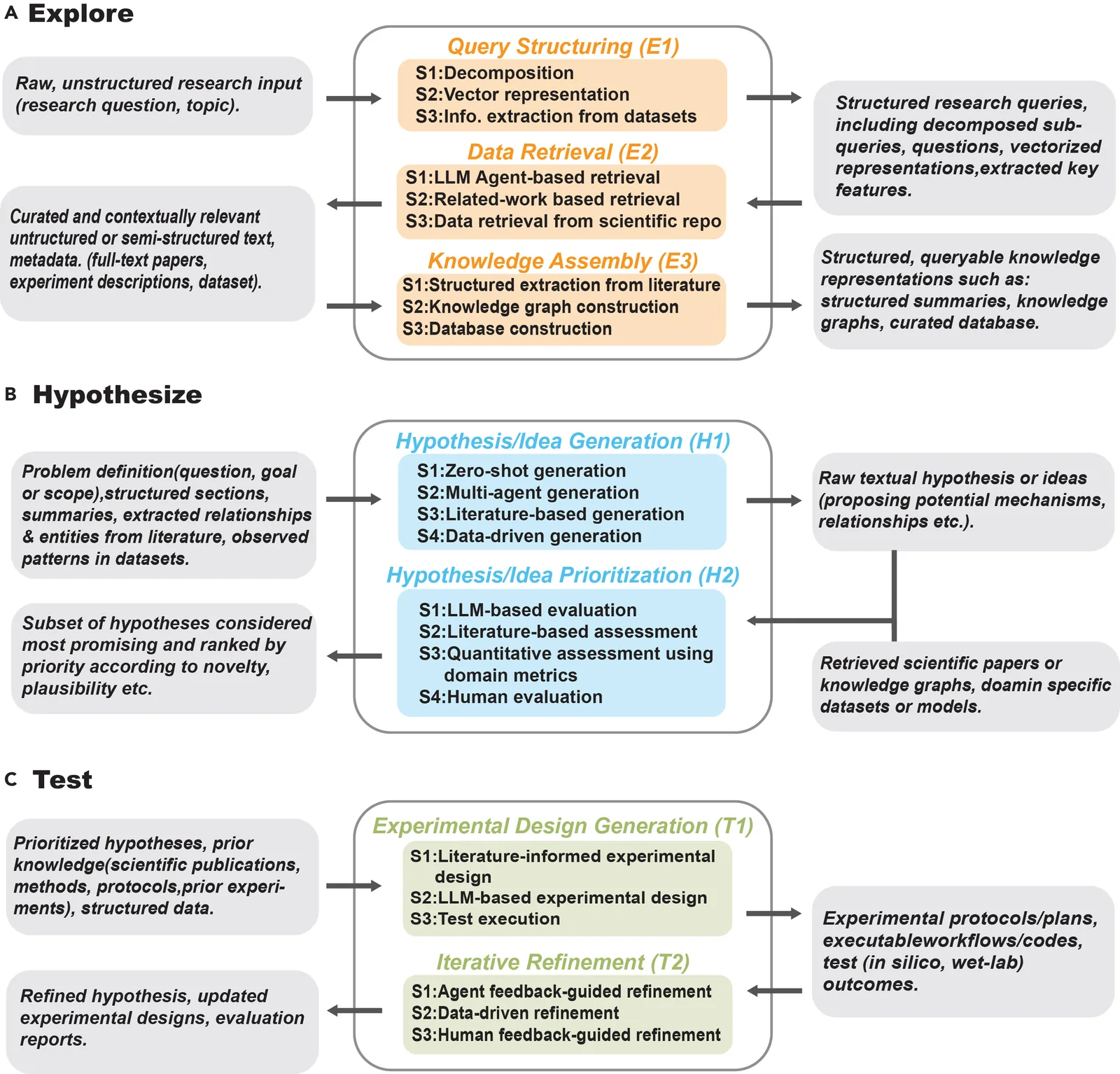
Inputs and outputs associated with each substage of the EXHYTE workflow (A) Explore: structures research questions, retrieves relevant scientific information, and assembles it into usable knowledge representations. (B) Hypothesize: generates candidate hypotheses from literature and data and then evaluates and prioritizes them. (C) Test: converts prioritized hypotheses into experimental designs and iteratively refines them using test outcomes and feedback.

**Figure 4:**
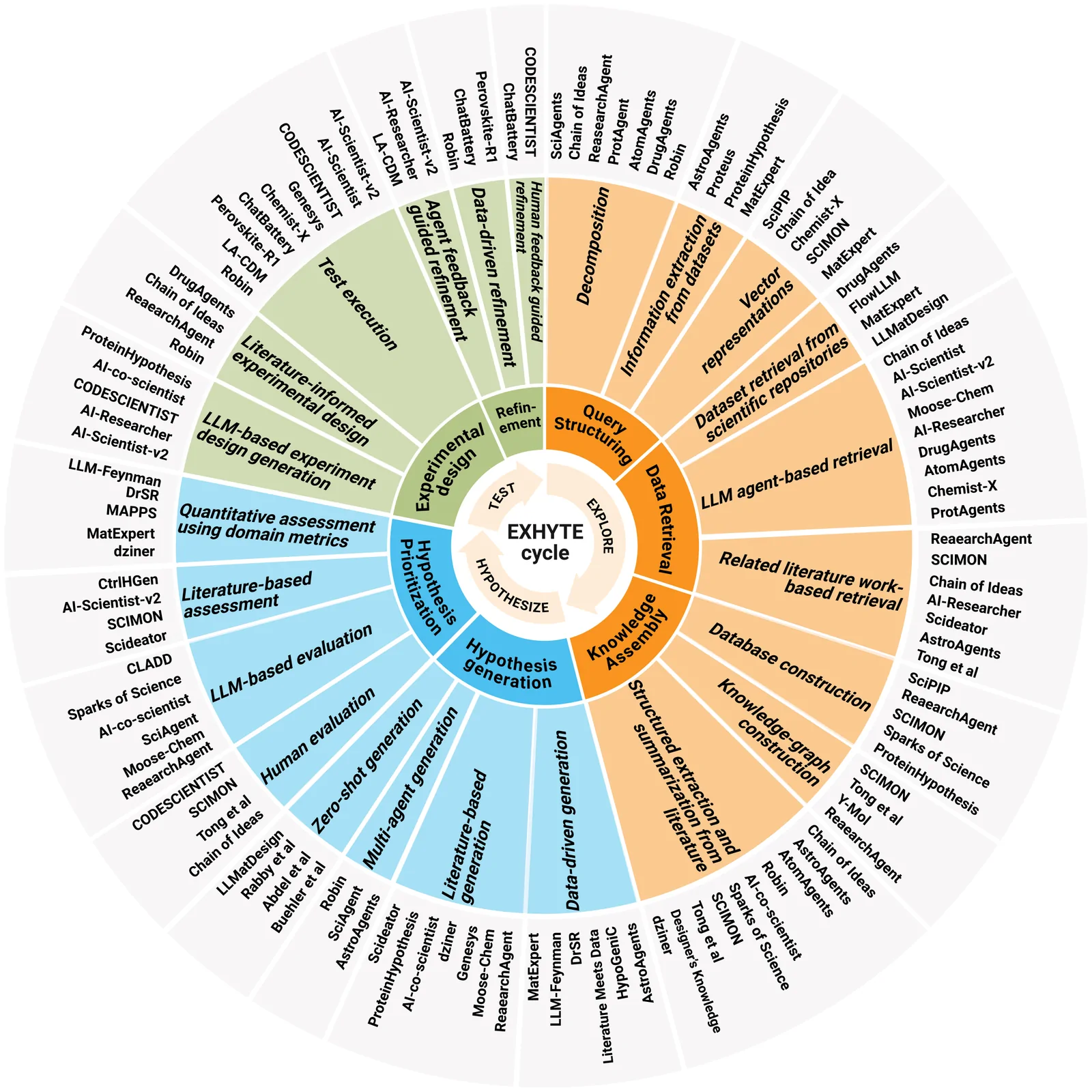
AI methods across the EXHYTE workflow and substages

*E1-S1: Decomposition:* Decomposition transforms complex research queries into tractable sub-queries/questions or tasks that enable focused literature/dataset retrieval, reasoning, and solution design using LLMs. Different systems operationalize decomposition at various levels. Chain of Ideas^24^ decomposes an initial research topic into multiple sub-queries that capture diverse perspectives on the same problem, SCI-IDEA^13^ extracts key phrases that represent the underlying research focus of a scientist’s query, Assay2Mol^25^ identifies keywords from user-provided text descriptions of target proteins or phenotypes, Robin^26^ formulates a series of general questions based on the name of the target disease that the scientist inputs into the system, and DrugLLM^27^ decomposes molecules into structural groups to facilitate reasoning about chemical properties.

Building on this principle, several systems employ LLM-based agents to decompose complex user inputs, whether research queries or design tasks, into smaller, manageable sub-tasks and to generate detailed, step-by-step workflow plans. General-purpose frameworks such as ResearchAgent,^18^ AI-Researcher,^28^ SciAgent,^29^ Zhou et al.,^30^ and domain-specific systems such as ProtAgent (protein design),^31^ AtomAgents (alloy design),^32^ MatExpert (material discovery),^4^ and DrugAgents (drug discovery)^33^ all follow a similar decomposition pipeline.

*E1-S2: Vector representations:* Encoding user inputs as vector representations enables efficient retrieval, semantic similarity search, and context-aware reasoning. Frameworks such as Chain of Ideas,^24^ SciPIP,^34^ SCI-IDEA,^13^ SCIMON,^35^ and IRIS^14^ transform research queries, questions, and background knowledge into embeddings to support retrieval of relevant scientific literature.

In domain-specific applications, Chemist-X^1^ encodes molecules as SMILES strings to retrieve reaction conditions for target products, while DrugMCTS^21^ identifies structurally similar molecules using vectorized molecular representations. Assay2Mol^25^ embeds protein target descriptions and phenotypic data for BioAssay retrieval, and MatExpert^4^ employs dual encoders with one for property descriptions and another for structural representations to improve reference material retrieval. Collectively, these systems show how vector representations encode scientific knowledge in a machine-interpretable form, enabling efficient semantic retrieval and context-aware scientific discovery.

*E1-S3: Information extraction from datasets:* Extracting key features and annotations from structured datasets enables automated systems to generate empirical inputs for downstream tasks such as model training, hypothesis formulation, and idea generation. AstroAgents,^36^ for instance, employs a “data analyst agent” to identify polycyclic aromatic hydrocarbon (PAH) patterns from user-provided mass spectrometry data, thereby generating testable hypotheses in astrobiology. Proteus^37^ integrates bioinformatics tools to uncover biological relationships through statistical analyses, while Qu et al.^37^ further identify pairs of biological entities (e.g., protein, clinic) to define research directions from multi-omics datasets. ProteinHypothesis^38^ retrieves protein sequence descriptions, structural features, and physicochemical properties to inform LLMs about sequence-structure-function relationships during hypothesis generation. In drug discovery, DrugMCTS^21^ extracts molecular properties and annotations from DrugBank^22^ and KIBA,^23^ while MatExpert^4^ extracts chemical formulas and physical properties (e.g., formation energy and elemental composition) from databases such as NOMAD^39^ and Materials Project^40^ to generate candidate crystal structures. LLM-Feynman^6^ automates feature selection and iterative refinement to construct high-quality feature sets, which are subsequently used in symbolic regression to derive interpretable physical equations. Together, these systems illustrate how information extraction transforms raw datasets into structured, semantically meaningful inputs that drive empirical reasoning and hypothesis development across scientific domains.

##### E2: Data retrieval

Retrieving literature and datasets is a foundational step in scientific discovery, ensuring that exploration remains grounded in comprehensive and relevant knowledge. Guided by user inputs, this E2 step compiles scientific publications and datasets that support informed hypothesis exploration and validation. We identified three key strategies that capture how systems retrieve information through LLM-based agents, related-work networks, and scientific repositories, respectively (Figures 3A and 4).

*E2-S1: LLM agent-based retrieval:* LLM agents now routinely query and curate literature and datasets. General-purpose frameworks such as Chain of Ideas^24^ and Graph of AI Ideas^41^ use LLMs to generate multiple research queries from an initial topic, capturing diverse conceptual angles. SciAgents^29^ and AI-Scientist,^42^ along with AI-Scientist-v2,^12^ retrieve literature via the Semantic Scholar API for novelty checks and evidence gathering.

LLM agents are also tasked with prioritizing the most relevant papers. IRIS^14^ introduces a retrieval agent that issues targeted queries answered by the specialized “AI2 Scholar QA” API. Moose-Chem^2^ generates titles from background papers to retrieve inspirational works. AI-Researcher^28^ designs a “knowledge acquisition agent” that, given reference papers, retrieves additional papers and code repositories from scientific databases.

In domain-specific settings, retrieval is coupled with scientific reasoning. DrugAgent^33^ employs an “instructor agent” to query domain libraries and translate abstract ideas into executable code via domain-specific examples. AtomAgents^32^ integrates a specialized knowledge-retrieval tool that extracts material properties directly from published papers. Chemist-X^1^ produces executable code to interrogate repositories such as PubChem for chemical literature and molecular datasets, while ProtAgents^31^ retrieves specific data points (e.g., Protein DataBank [PDB] identifiers) for protein design. In materials discovery, AutoMAT^15^ uses LLMs, together with user-defined property targets and constraints, to consult reference handbooks (e.g., titanium alloy databases) and identify candidates meeting elemental criteria.

*E2-S2: Related-work-based retrieval:* Citation networks and semantic similarity are widely used to link related works and surface new directions. ResearchAgent,^18^ SCIMON,^35^ HypER,^20^ and Chain of Ideas^24^ expand from an anchor paper by retrieving both citing and cited works to capture the evolution of research themes; HypER further adds LLM-based relevancy scoring. AI-Researcher^28^ incorporates multi-step reference selection for each target paper, including citation-pattern analysis (frequency), context analysis (influence on methods/theory/experiments), evidence collection (textual support), and an integrated impact score. SciPIP^34^ combines keyword, co-occurrence, and semantics-based retrieval and then applies a cluster-based selection of representative papers. SCI-IDEA^13^ queries academic sources using key phrases drawn from a researcher’s work and similar literature. Scideator^43^ retrieves multiple analogs per source paper, uses an LLM to generate “purpose-mechanism” pairs, and then issues queries per pair to find additional relevant papers. Domain-focused approaches tailor queries to specialized terms. For example, ProteinHypothesis^38^ uses domain-specific keywords (e.g., “Protein Science”) to retrieve metadata (titles, abstracts, and links); AstroAgents^36^ selects relevant papers and books to provide astrobiology context; and Tong et al.^44^ require the presence of terms such as “psychol,” “clin psychol,” and “biol psychol” in titles or abstracts for psychology/neuroscience. Manual and semi-automatic curation complement these methods via direct selection from leading databases (e.g., Sparks of Science,^45^ Perovskite-R1,^46^ Budget AI Researcher,^47^ Lee et al.,^16^ Quilodrán-Casas et al.,^48^ Cheng et al.,^49^ Ciucua et al.^50^).

*E2-S3: Dataset retrieval from scientific repositories:* Datasets provide the empirical foundation for analysis, model training, and validation. In drug discovery, DrugMCTS^21^ queries databases to retrieve molecules structurally similar to a query compound, associated protein interactors, and binding-pocket information parsed from PDB files to improve LLM interpretability. DrugAgent^33^ retrieves biological datasets from the Therapeutics Data Commons (TDC), which offers structured, AI-ready datasets and benchmarks across drug development stages. In materials discovery, FlowLLM^51^ retrieves inorganic crystalline materials from the Materials Project for LLM fine-tuning, and Perovskite-R1^46^ assembles drug-like compounds from a comprehensive library to enhance generalization across chemical space. MatExpert^4^ and LLMatDesign^5^ access NOMAD^39^ and the Materials Project^40^ for reference materials used in training, evaluation, and modification workflows. Zhou et al.^30^ retrieve crystal structures with compositions or reduced formulas similar to a query from structural databases to identify promising crystal prototypes.

##### E3: Knowledge assembly

The knowledge-assembly stage transforms unstructured scientific text into structured, queryable representations that enable efficient reasoning during discovery. The existing literature typically employs three strategies: structured extraction and summarization from literature, knowledge-graph construction, and database construction for large-scale retrieval and synthesis (Figures 3A and 4).

*E3-S1: Structured extraction and summarization from literature:* To make literature readily usable for idea and hypothesis generation, systems extract and summarize standardized sections (e.g., titles, abstracts, methods, and results). SciPIP,^34^ Chain of Ideas,^24^ SCI-IDEA,^13^ and AstroAgents^36^ implement pipelines that segment papers into consistent fields. Papers like SciPIP,^34^ AtomAgents,^32^ Robin,^26^ Literature Meets Data,^52^ AI-co-scientist,^53^ and IRIS^14^ further produce concise synopses of key findings. Sparks of Science^45^ uses LLMs to extract each paper’s conventional assumption and innovative approach alongside a concise insight summary from abstracts. Several systems, including SCI-IDEA,^13^ Scideator,^43^ SCIMON,^35^ and Chen et al.,^54^ extract facets such as objectives, methods, evaluation, and future work. Domain-tailored extraction includes causal relations in psychology (Tong et al.^44^), question-answer pairs for perovskite precursors (Perovskite-R1^46^), frequent-topic mining to guide retrieval (Lee et al.^47^), structured tables of target properties, related structures, and linking mechanisms (Beyond Designer’s Knowledge^55^), parsing mechanical properties and compositions to propose alloys (AutoMAT^15^), and sentence-level evidence selection for property-optimization queries (dziner^56^).

*E3-S2: Knowledge graph construction:* Knowledge graphs (KGs) encode entities and relations to support structured reasoning and discovery. SCIMON^35^ represents tasks, methods, materials, and metrics as nodes, with “used-for” edges to ground generated ideas. Tong et al.^44^ construct causal KGs in psychology, where cause and effect pairs are directed nodes with attributes (interpretations) and relation types (causal vs. correlational). Y-Mol^57^ builds a biomedical KG with interactions among drugs, genes, and diseases (drug-target, drug-drug, and disease-gene). ResearchAgent^18^ assembles an entity-centric store from co-occurrences in literature to expose cross-domain connections (topics, keywords, individuals, and events). SciPIP^34^ links papers to LLM-extracted keywords in a paper-keyword graph for efficient keyword-based retrieval. Graph of AI Ideas^41^ constructs a paper-level graph using structured quadruples (paper, citation position, citation semantics, and paper), where citation position captures reference importance and citation semantics defines the purpose of each citation.

*E3-S3: Database construction:* Curated databases consolidate heterogeneous resources into structured repositories for retrieval, analysis, and synthesis. SciPIP^34^ builds a literature database from top venues over the past decade, storing key sections and re-summarizing each paper into a quintuple (keywords, background, ideas, concise methods, and core references), with background/ideas encoded as embeddings. ResearchAgent^18^ extracts entities from titles/abstracts (e.g., post-2023 Semantic Scholar) and encodes their co-occurrences in a sparse matrix. HypER^20^ compiles temporal reasoning-chain datasets linked to related papers for effective reasoning over scientific literature. SCIMON^35^ builds corpora from the ACL Anthology and PubMed, organizing the papers with extracted (background, target) pairs, seed terms, and their relations. Sparks of Science^45^ constructs the “HypoGen” dataset by extracting assumptions, innovations, and core insights from abstracts. ProteinHypothesis,^38^ Lee et al.,^47^ and Cheng et al.^49^ store vector embeddings of papers from arXiv and major conferences (e.g., NeurIPS and ICLR) to support scalable retrieval.

#### The Hypothesize stage

##### H1: Hypothesis/idea generation

Hypothesis and idea generation mark the transition from structured knowledge to discovery, synthesizing literature, data, and reasoning strategies into creative, testable hypotheses. We identify four strategies commonly employed in the literature, including zero-shot generation, multi-agent generation, literature-based generation, and data-driven generation (Figures 3B and 4).

*H1-S1: Zero-shot generation:* Zero-shot generation relies on the internal knowledge encoded in LLMs, thus enabling idea/hypothesis creation without explicit external context—useful when domain-specific training data are limited. Frameworks illustrating this approach include LLMatDesign,^5^ Buehler et al.,^58^ Tang et al.,^59^ Yin et al.,^60^ Bystronski et al.,^61^ Abdel et al.,^62^ Rabby et al.,^63^ Guo et al.,^64^ Pu et al.,^17^ Liu et al.,^65^ and Manning et al.^66^

*H1-S2: Multi-agent generation:* Multi-agent frameworks build specialized agents, coordinated under an orchestrator agent to produce and refine hypotheses. AstroAgents^36^ employs a hierarchical design, where a “data analysis” agent first uncovers key patterns and trends and a “planner” agent delegates distinct data segments to multiple “scientist” agents for in-depth exploration and investigation; finally each scientist agent operates within its assigned domain to produce hypotheses, which are consolidated by an “accumulator” agent that de-duplicates and integrates the results. The Virtual Lab^67^ uses a “principal investigator” agent to spawn a team of domain-specific scientist agents and a “scientific critic” agent that, through structured meetings, generate and iteratively refine candidate hypotheses and designs (e.g., SARS-CoV-2 nanobody candidates). In the KG-grounded SciAgents,^29^ an “assistant” agent drafts an initial subgraph to contextualize the idea, an “ontologist” agent defines nodes and interprets edges, and two scientist agents iteratively draft, elaborate, and critique detailed hypotheses. Robin^26^ specializes in biomedicine and designed an agent (“Crow”) to identify causal disease mechanisms and suitable *in vitro* models and another agent (“Falcon”) to conduct deep literature review to evaluate therapeutic candidates. In chemical discovery, DrugMCTS^21^ coordinates multiple sequential agents to incrementally build reasoning paths for protein target identification, where a “molecule-analysis” agent produces natural-language molecular reports, a “molecule-selection” agent filters candidates, an “interaction-analysis” agent explores molecule-protein interactions, and a “decision” agent synthesizes upstream findings to predict promising targets.

*H1-S3: Literature-based generation:* Literature-driven approaches retrieve, synthesize, and recombine knowledge from scientific texts to propose hypotheses. ResearchAgent^18^ and Moose-Chem^2^ provide titles/abstracts of various related papers as context for LLMs. Genesys^49^ formulates new LLM design ideas by modifying parent designs from the Knowledge Engine (KE), which integrates the curated literature with code and external sources. Similarly, dziner^56^ generates modifications based on retrieved material-design guidelines mined from unstructured text (e.g., scientific literature and the Internet). Another common strategy is summarization-based synthesis, where relevant sections are distilled and restructured before prompting the LLM (e.g., AI-co-scientist,^53^ SciPIP,^34^ ProteinHypothesis,^38^ and Perovskite-R1^46^). Another approach is facet recombination, in which facets (e.g., purpose and mechanism) are recombined to surface gaps and generate ideas. For example, Scideator^43^ constructs analogies from purpose-mechanism pairs across papers; SCI-IDEA^13^ analyzes similar facets to detect patterns, inconsistencies, and unexplored areas; Liu et al.^55^ and Lee et al.^47^ synthesize interdependencies among mechanisms or topics not explicitly linked in the source literature; and CODESCIENTIST^19^ explores combinatorial idea generation via genetic operators (e.g., crossover, mutation). A complementary line of work is a reasoning chain-based generation, which reconstructs explanatory pathways from the noisy/fragmented literature. HypER,^20^ Sparks of Science,^45^ and Graph of AI Ideas^41^ fall into this line and identify valid chains in the literature graphs and use them as scaffolds for evidence-grounded hypotheses. Finally, KGs are proposed to structure scientific concepts and relations for systematic hypothesis generation. For example, Tong et al.^44^ employ link prediction algorithms on causal KGs to identify high-probability causal relationships among previously unconnected concepts; Lee et al.^16^ integrate biochemical drug-drug relationships from a KG to interpret the network structure and then pass these summaries to a prediction agent; Gao et al.^68^ generate plausible logical hypotheses from KG entity sets.

*H1-S4: Data-driven generation:* Structured datasets provide a complementary source, where data instances and patterns can guide hypothesis formation. One group of systems detects patterns and supplies them as context to the LLM. In AstroAgents,^36^ a “data analyst” agent examines mass spectrometry to uncover PAH distributions and alkylation patterns, highlighting unexpected findings. ProteinHypothesis^38^ analyzes sequence motifs, secondary-structure correlations, and functional-site patterns from experimental data. Proteus^37^ runs targeted statistical analyses over multi-omics data using bioinformatics tools, recording quantified relationships between biological entities (e.g., proteins, transcripts, and variants) and clinical features (e.g., survival time and tumor stage) as edges in a “conclusion graph” that then grounds LLM hypothesis generation. Biomni,^69^ a general-purpose biomedical agent, follows a similar approach but composes its analytical pipeline dynamically through code execution by an LLM agent, rather than relying on a fixed analyst module. Another line seeds the LLM with few-shot data instances: HypoGeniC,^70^ Literature Meets Data,^52^ and Hypotheses for Inductive Reasoning^71^ provide small sets of (input, label) pairs to induce initial hypotheses. In DrSR,^72^ ChatSR,^73^ and LLM-Feynman,^6^ observational pairs guide LLMs in analyzing structural relationships, which are then used by symbolic regression to produce interpretable formulas. Zheng et al.^74^ discover molecular patterns using structural/chemical features and then use them to predict molecular properties. Liu et al.^75^ retrieve domain-specific drug data as demonstrations for LLM to perform drug-editing tasks. In materials science, MAPPS^30^ queries structural databases for crystal prototypes with compositions/reduced formulas similar to a query, using them to generate initial candidate structures; MatExpert^4^ fine-tunes a small LLM on curated transition pathways and target materials from NOMAD to generate plausible transition pathways. In healthcare and clinical design, Zhu et al.^76^ and Bani et al.^77^ fine-tune LLMs on patent-title pairs or clinical datasets containing various clinical scenarios and ground-truth diagnoses to enable new design ideas and hypotheses.

##### H2: Hypothesis or idea prioritization

Hypothesis prioritization focuses analytical and computational resources on the most promising ideas by evaluating their novelty, plausibility, feasibility, and potential impact. We identify four common strategies: LLM-based evaluation, literature-based assessment, quantitative assessment using domain metrics, and human evaluation (Figures 3B and 4).

*H2-S1: LLM-based evaluation:* LLM agents can act as evaluators, ranking ideas/hypotheses on clarity, feasibility, novelty, plausibility, consistency, and impact. Examples include ResearchAgent,^18^ AstroAgents,^36^ SciAgents,^29^ AI-co-scientist,^53^ Sparks of Science,^45^ Moose-chem,^2^ Tong et al.,^44^ PROTEUS,^37^ Rabby et al.,^63^ ProteinHypothesis.^38^ Robin^26^ uses an LLM judge to rank drug candidates by scientific rationale, pharmacological profile, and methodology of the supporting literature. AstroAgents^36^ uses a critic agent to assess the alignment between hypotheses and observational data. CLADD^16^ evaluates drugs by predicting toxicity from a molecule’s chemical representation (SMILES), inferring likely protein targets (mechanism of action) from an LLM-agent-generated report on the query molecule, and categorizing molecular properties from a generated textual description concatenated with the SMILES representation. DrugMCTS^21^ extends this by integrating LLM-based reward functions within a Monte Carlo Tree Search framework to score molecular-protein interactions using structural (binding pocket) and literature-derived contextual evidence. ChatBattery^78^ uses a “search” agent to verify novelty by querying the Materials Project database and a “retrieval” agent to identify similar lithium-based compounds from the Inorganic Crystal Structure Database (ICSD), guiding subsequent hypothesis revisions. Liu et al.^55^ evaluate materials design hypotheses based on the alignment between the stated materials design goals and the content of the hypothesis. LLM-Feynman^6^ employs an LLM to assign an “interpretability score” to its own generated formulas, quantifying their physical and chemical meaning, and ProtAgents^31^ assesses success in generating proteins with desired secondary structures.

*H2-S2: Literature-based assessment:* To ensure that generated hypotheses are both novel and scientifically grounded, many systems incorporate literature-based assessment as a critical step. This process involves retrieving relevant prior work, evaluating semantic similarity, and determining whether the proposed idea contributes to new insights. Systems such as Scideator,^43^ HypER,^20^ SCIMON,^35^ AI-scientist,^42^ AI-Scientist-v2,^12^ AstroAgents,^36^ Graph of AI Ideas,^41^ SCI-IDEA,^13^ SciAgents,^29^ Budget AI Researcher,^47^ Cheng et al.,^49^ and Lee et al.^47^ deploy an LLM-based “Novelty Checker,” which assesses the idea’s novelty against relevant papers. HypER^20^ evaluates whether the rationale and hypothesis track the logic and chronology of literature chains. CtrlHGen^68^ measures the alignment between hypothesis conclusions and the observed entities in the KG using multiple similarity metrics. These metrics are incorporated into the reward function that guides hypothesis generation.

*H2-S3: Quantitative assessment using domain metrics:* Quantitative evaluation using domain-specific scientific metrics and computational models provides an objective measure of hypotheses. dziner^56^ calculates “synthesizability” (e.g., synthetic accessibility score and quantitative estimate of drug-likeness [QED score]) using a PubChem-scale molecule dataset and specialized tools. The effectiveness of a proposed modification is then evaluated with domain-expert predictive models, and the LLM ultimately decides whether to accept or reject the modification based on these quantitative results. Matexpert^4^ evaluates generated material structural validity, chemical feasibility, and stability using quantitative and physics-informed metrics. ChatBattery^78^ verifies the stability and properties of the top-ranked cathode candidates using a machine learning surrogate model (MACE-MP) that predicts the energies and forces of the sampled structures. MAPPS^30^ evaluates crystal structures for structural quality, thermodynamic stability, novelty, and accuracy. Liu et al.^75^ compare edited molecules with desired properties and quantify binding affinity for peptide editing and secondary structural content for protein secondary structure editing relative to desired targets. LLMatdesign^5^ predicts properties via a machine learning property predictor (MLPP) after a machine learning force field (MLFF)-based relaxation of modified material structures, declaring success when the predicted properties of the new material align with target specifications. In symbolic reasoning and equation discovery, DrSR^72^ and ChatSR^73^ iteratively improve model output by assessing generated equations using numerical fitness scores and domain performance metrics. LLM-Feynman^6^ provides a comprehensive quantitative evaluation framework using the coefficient of determination and mean absolute error (MAE) for regression tasks, and accuracy, precision, recall, F1 score, and cross-entropy loss for classification-based symbolic modeling.

*H2-S4: Human evaluation:* Expert review complements automated scoring with contextual judgment and domain nuance. Chain of Ideas,^24^ Tong et al.,^44^ AstroAgents,^36^ SciPIP,^34^ SciMON,^35^ CODESCIENTIST,^19^ SCI-IDEA,^13^ Literature Meets Data,^52^ ChatBattery,^78^ Rabby et al.,^63^ and HypER^20^ incorporate expert feedback to refine, rank, or filter hypotheses.

#### The Test stage

##### T1: Experimental design generation

Experimental design generation translates top-ranked ideas and hypotheses into verifiable experimental plans, creating a systematic path to evaluate scientific propositions. It comprises three common strategies: literature-informed design, LLM-based design generation, and test execution (Figures 3C and 4).

*T1-S1: Literature-informed experimental design:* This strategy leverages prior studies and domain references to ground experimental plans in established scientific practices. Robin^26^ selects top-ranked “*in vitro*” models from the literature review to define therapeutic testing strategies and generates concise experimental outlines. ResearchAgent^18^ synthesizes problem information, methods, and existing designs from the literature into new protocols that emphasize clarity, robustness, reproducibility, validity, and feasibility. Chain of Ideas^24^ uses few-shot prompting with examples of prior experiments, together with user-provided ideas and key entities, to guide design generation. Similarly, DrugAgent^33^ constructs executable code from curated documentation, compiled from widely used libraries and models from the literature. Together, these approaches ensure that the designs are evidence-based while adaptable to scientific contexts.

*T1-S2: LLM-based experiment design generation:* Beyond literature grounding, systems increasingly rely on LLM reasoning to autonomously generate experimental designs, often producing executable workflows or code for validation. AI-Scientist-v2^12^ employs an “Agentic Tree Search algorithm” to systematically explore hypotheses during the experimentation phase. For each node in the experimental pathway, an LLM proposes a concrete experimentation plan and the Python code to implement it. Similarly, AI-Researcher,^28^ Lee et al.,^71^ Lui et al.,^65^ Cheng et al.^49^ transform research analyses and development plans into executable code via LLM agents. In physics-based discovery, Zhou et al.^30^ invoke tools from a physics toolbox to translate workflow steps into executable, physically grounded Python code. CODESCIENTIST^19^ uses a “planner” agent that ingests the idea, expert comments, and a codeblock library to produce a concrete plan and required codeblocks. AI-co-scientist^53^ features a “generation” agent that proposes experimental protocols for downstream validations. In a biomedical experimental setting, ProteinHypothesis^38^ assesses the experimental feasibility of hypotheses and maps the hypotheses to laboratory techniques, while Chemist-X^1^ generates control scripts for an agent to operate wet-lab platforms for validation. Brunnsåker et al.^79^ likewise uses LLM agents to turn a hypothesis into an experimental plan and auto-formalize it into a validated, machine-readable protocol for an automated yeast platform.

*T1-S3: Test execution:* This strategy validates LLM-generated hypotheses via *in silico* or wet-lab experiments, closing the discovery loop by determining the real-world feasibility and accuracy of these hypotheses. In Robin,^26^ the experimental strategies are proposed for validating therapeutic candidates by identifying a relevant disease mechanism and *in vitro* models and executed by human researchers in an iterative lab-in-the-loop framework. In clinical hypothesis refinement, LA-CDM^77^ uses a decision agent to request additional diagnostic tests to reduce uncertainty. Perovskite-R1^46^ implements recommended protocols and fabricates complete solar cell devices to assess the efficacy of the selected additives. ChatBattery^78^ synthesizes and tests cathode candidates through wet-lab experiments. Chemist-X^1^ validates generated reaction conditions through fully automated wet-lab experiments under LLM supervision. Brunnsåker et al.^79^ executes auto-formalized protocols on a fully automated cell-culture and mass spectrometry platform. Abdel et al.^62^ test LLM-proposed drug combinations in the lab.

Execution also includes computational validation. Tang et al.^59^ apply regression-based statistical models to test hypotheses about LLM’s explanatory power for societal outcomes. Genesys^49^ evaluates the proposed language model design by verifying code correctness, conducting training-time experiments, and measuring success via a multi-scale fitness function. CODESCIENTIST^19^ follows an iterative generate-execute-reflect debugging loop, while AI-Scientist^28^ and AI-Scientist-v2^12^ test LLM-generated experiment prototypes by running associated Python code to test conceptual validity.

*T2: Iterative refinement:* Iterative refinement improves the quality and feasibility of hypotheses, ideas, and experimental designs by incorporating feedback from agents, data, and experts, ensuring responsiveness to new information and evaluations. There are three common strategies: agent feedback-guided refinement, data-driven refinement, and human feedback-guided refinement (Figures 3C and 4).

*T2-S1: Agent feedback-guided refinement:* Systems employ feedback loops in which LLM-based agents iteratively revise hypotheses, code, and designs to improve feasibility and correctness. AI-Scientist-v2^12^ refines algorithm nodes through agent feedback by evaluating “parent nodes” outcome, where buggy parents trigger debugging using child nodes, while non-buggy parents prompt experimental refinement. Key metrics (e.g., loss values and training data) are logged and visualized, and a vision language model (VLM) then critiques plots for label clarity, legend completeness, and data accuracy. Nodes flagged by the VLM are marked buggy, whereas validated nodes are retained, enabling continuous, feedback-driven improvement. Similarly, AI-Researcher^28^ utilizes an “advisor” agent to propose actionable refinement spanning implementation details, validation studies, visualizations, and comparative analyses. In clinical settings, the “hypothesis” agent in LA-CDM^77^ updates diagnoses and confidence estimates as patient-state information evolves, repeating until a final diagnosis is produced.

*T2-S2: Data-driven refinement:* Data-driven refinement keeps hypotheses, models, and experimental plans empirically grounded by updating them with new computational and experimental evidence. In Robin,^26^ human scientists upload raw or semi-processed data (e.g., flow cytometry and RNA sequencing [RNA-seq] data), which are processed by the “Finch” analysis agent with the requested analysis to generate statistical tests and differential gene expression plots. Robin also distills actionable insights from the analysis results and proposes follow-up assays. Fully automated systems such as Chemist-X^1^ execute LLM-generated reaction conditions with closed-loop wet-lab experiments and quantify reaction outcomes (e.g., the product yield using high-performance liquid chromatography). Abdel et al.^62^ validate LLM-generated drug combinations experimentally and compute highest single agent (HSA) synergy scores to quantify performance. In materials science, ChatBattery^78^ characterizes physical/structural performance with power conversion efficiency (PCE), film quality, and stability. Perovskite-R1^46^ assesses the efficacy of the selected additives by fabricating complete solar cell devices and evaluating metrics such as PCE, film quality, and long-term device performance. In social science, Tang et al.^59^ convert natural language hypotheses into interpretable embeddings used as explanatory variables in a linear regression model to predict societal outcomes. Coefficients are tested for significance, non-significant hypotheses are pruned, and the feedback guides subsequent LLM proposals.

*T2-S3: Human feedback-guided refinement:* Expert review adds domain-specific judgment that complements automated evaluations. In ChatBattery,^78^ human researchers revise hypotheses after examining experimental outcomes, aligning hypotheses with objectives and constraints when results indicate limited validity or feasibility. CODESCIENTIST^19^ generates concise reports indicating whether each hypothesis was confirmed, rejected, or inconclusive, and final expert screening substantially narrows the candidate set.

### Integrated EXHYTE workflows: Case studies

The previous sections examined how individual studies address specific stages and substages of the EXHYTE framework. This stage-by-stage analysis provides a granular view of methodological contributions, but it does not fully show how these components are combined in existing AI-for-science systems. To illustrate workflow integration in practice, we examine two published systems from the surveyed literature: AI-Researcher^28^ and AI Co-Scientist.^53^ These systems were selected as case studies because they connect multiple EXHYTE functions within relatively complete workflows while differing in modality, degree of autonomy, and human involvement. AI-Researcher illustrates an *in silico* workflow centered on literature, code, algorithm design, and computational evaluation, whereas AI Co-Scientist illustrates a scientist-in-the-loop workflow that links LLM-based reasoning with wet-lab validation. We use these examples not as systems proposed by us but as representative cases for analyzing how existing methods instantiate the EXHYTE workflow abstraction.

#### AI-Researcher: An *in silico* workflow

The goal of the AI-Researcher system is to design a complete research pipeline with minimal human intervention capable of systematically targeting the conceptual frontiers of science, deliberately seeking conceptual gaps, contradictory findings, and emerging patterns across literature and implementations. It begins with a curated collection of approximately 15–20 reference papers, along with optional datasets, which form the foundation of knowledge for the system. These sources mirror the intellectual basis of a target publication, providing the conceptual and methodological context from which new discoveries emerge.

##### Explore

During the exploration stage, AI-Researcher establishes the technical and conceptual groundwork for autonomous algorithm design. This process is jointly managed by the *knowledge acquisition agent* and the *resource analyst agent*, supported by their respective sub-agents. The *resource analyst agent* decomposes the research objective into atomic academic concepts, which are fundamental, indivisible elements requiring targeted investigation. Guided by these concepts, the *knowledge acquisition agent* retrieves relevant papers and code repositories from scientific databases, applying multi-criteria filtering to select high-quality sources (E2-S1/S2). The *paper analyst* sub-agent extracts mathematical formulations from LaTeX files using a retrieval-augmented generation (RAG) approach (E3-S1), while the *code analyst* identifies corresponding code implementations, establishing explicit bidirectional mappings between theory and code (E3-S3). The resulting structured report provides the empirical and theoretical foundation for downstream reasoning.

##### Hypothesize

Building on the knowledge assembled during exploration, the *idea generator* identifies conceptual gaps, contradictory findings, and emerging patterns across literature and implementations (H1-S3). It employs a Divergent-Convergent Discovery Framework: the divergent phase produces multiple conceptually distinct research directions, while the convergent phase evaluates them using criteria such as novelty, soundness, and transformative potential (H2-S1/S2). The *advisor agent* provides expert-style feedback linking theoretical concepts to implementation plans, embodying the human-in-the-loop evaluation principle (H2-S4).

##### Test

The testing phase operationalizes selected hypotheses through experimental planning, implementation, and iterative refinement. The *plan agent* generates a comprehensive development plan, including dataset selection, training/testing methodology, and evaluation metrics (T1-S1). The *code agent* translates this plan into executable implementations, verifying dataset usage and experimental consistency (T1-S2). Results are analyzed by the *experiment analysis agent*, which recommends code modifications and further experimentation (T2-S1). Finally, the *automated documentation agent* revises the manuscript using an academic checklist to ensure clarity, reproducibility, and completeness (T2-S3). Together, these stages demonstrate a continuous feedback loop linking hypothesis generation to empirical validation within a coherent *in silico* research pipeline.

#### AI Co-Scientist: A wet-lab-integrated workflow

The primary goal of the AI Co-Scientist is to accelerate the process of scientific discovery by uncovering new, original knowledge operating within a “scientist-in-the-loop” collaborative paradigm. AI Co-Scientist implements the EXHYTE framework with physical experimentation by coupling LLM reasoning with wet-lab validation. The system accepts multiple input types from the human scientist, most critically a research goal, which may range from concise statements to long documents containing numerous prior publications.

##### Explore

Upon receiving the research goal, AI Co-Scientist parses it into a *research plan configuration* specifying desired outputs, constraints, and evaluation criteria (E1-S1). This configuration serves as a blueprint for downstream hypothesis generation. The *generation agent* then explores relevant literature via web search and retrieval tools (E2-S1/S2) to identify promising directions for new findings.

##### Hypothesize

The *generation agent* grounds its reasoning in summarized prior work and produces hypotheses and research plans (H1-S3). A *reflection agent* simulates a scientific peer-review process, critically examining each hypothesis for correctness, novelty, and evidential grounding (H2-S2). Quick reviews are followed by comprehensive evaluations that integrate external search and evidence retrieval to assess validity. For complex hypotheses, deep verification decomposes each claim into constituent assumptions, which are independently examined to detect invalid components. The *ranking agent* employs an Elo-style tournament framework to compare hypotheses through multi-turn debates, scoring them on novelty, correctness, and testability (H2-S1). The top-ranked hypotheses are iteratively refined by an *evolution agent* (T2-S1/S3).

##### Test

The testing stage connects LLM-generated hypotheses to real-world validation. AI Co-Scientist has been deployed in several biomedical domains, with wet-lab validation. Notably, the system generated the hypothesis that *chromosomal phage-inducible chromosomal islands* (cf-PICIs) *hijack phage tails to expand host range*, which was experimentally validated.^80^ This “tail-hijacking” mechanism revealed a previously unknown route for horizontal gene transfer in bacteria, advancing the understanding of antimicrobial resistance evolution. This case exemplifies how EXHYTE-integrated reasoning—spanning literature exploration, hypothesis generation, prioritization, and refinement—can produce experimentally verified discoveries.

Together, AI-Researcher and AI Co-Scientist illustrate how EXHYTE strategies interconnect across modalities. In both, *Explore* stages ground the system in literature and data; *Hypothesize* stages generate and prioritize testable ideas; and *Test* stages operationalize and refine them through computation or experimentation. These integrated workflows demonstrate the practical synthesis of the modular strategies surveyed earlier. This integration, however, is achieved through extensive orchestration. Both systems decompose the discovery pipeline into specialized, prompted agents connected by explicit control logic. This fragmentation is not inherently a weakness: separating roles improves interpretability, isolates failures, and allows targeted prompting and verification at each step. It also, however, introduces coordination overhead, numerous inter-agent interfaces that can drift or fail, and reproducibility challenges arising from accumulated orchestration logic. Much of this partitioning reflects the current engineering strategy for eliciting reliable behavior from general-purpose LLMs rather than an intrinsic requirement of scientific discovery; accordingly, newer general-purpose coding agents (e.g., Claude Code and Codex), whose explicit planning and execution phases can spawn sub-agents on demand, are beginning to collapse several of these hand-specified roles into fewer, more capable agents.

### Tools and datasets for building EXHYTE workflows

The preceding sections surveyed strategies for each EXHYTE stage. Here, we complement that view with the practical tools, APIs, and datasets that enable those strategies to operate (Tables 1 and 2). We group resources by their role in the framework and note where they most naturally plug into EXHYTE.

**Table 1:** AI tools and APIs supporting the EXHYTE workflow

Category	Tools and APIs
Literature and data retrieval APIs (E2)	Semantic Scholar,^81^ PubMed,^82^ arXiv,^83^ OpenAlex,^84^ PubChem,^85^ NASA ADS,^86^ and Materials Project^40^
Text parsing and normalization (E3)	PyPDF2,^87^ SciPDF,^88^ pymupdf4llm,^89^ S2ORC doc2json,^90^ RecursiveCharacterTextSplitter,^91^ and BeautifulSoup^92^
Embedding and representation (E2/E3)	OpenAI text embeddings,^93^ BERT,^94^ SciBERT,^95^ Sentence-BERT,^96^ BAAI/bge-large-en-v1.5,^97^ Google Generative AI Embeddings,^98^ jina Embeddings v.3,^99^ and ChemBERTa^100^
Similarity search and indexing (E2/H2)	FAISS^101^ and Tanimoto similarity^102^
Text mining and knowledge graph construction (E3)	BLINK,^103^ NER (Entity Linker),^104^ PL-Marker,^105^ SciCo,^106^ and SciSpaCy^107^

**Table 2:** Benchmark datasets across domains

Category	Subcategory	Dataset names
Drug discovery and bioactivity	drug-target interaction	DrugBank,^22^ KIBA,^23^ and DAVIS^108^
	large compound collections	ZINC^109^ and ChEMBL^110^
	protein-ligand docking	CrossDocked2020^111^
	clinical and patient data	MIMIC-CDM^112^
Materials science and chemistry	inorganic crystal structures	MP-20,^40^ NOMAD,^39^ and JARVIS-DFT^113^
	materials property prediction	Matbench^114^
	catalyst structures	OC20-S2EF 2M^115^
	chemistry hypothesis generation	TOMATO-Chem^2^
	superconducting materials	Superconductor Optimization (SPO)^17^
Multi-domain, NLP, and AI reasoning	binary classification and suitability	BUSINESS SHOES^116^
	deception detection	DECEPTIVE REVIEWS^117^
	popularity prediction	HEADLINE POPULARITY^118^ and TWEET POPULARITY^119^
	mental stress and persuasion	DREADDIT^120^ and PERSUASIVE PAIRS^121^
	systematic review and literature reasoning	RCT Summaries^122^ and HypoGen^45^

#### Core tools and APIs

##### Literature and data access (E2)

For scientific publications and metadata, commonly used resources include Semantic Scholar,^81^ PubMed,^82^ arXiv,^83^ OpenAlex,^84^ and NASA ADS.^86^ Chemical and materials data are typically sourced from PubChem^85^ and the Materials Project.^40^

##### Text parsing, extraction, and normalization (E3)

A variety of tools are employed for text parsing, extraction, and normalization. PDF and document content are commonly processed using PyPDF2,^87^ SciPDF,^88^ pymupdf4llm,^89^ and S2ORC-doc2json,^90^ while RecursiveCharacterTextSplitter^91^ is used for document chunking. HTML content is parsed with BeautifulSoup.^92^ For entity extraction and normalization, tools such as BLINK,^103^ the NER^104^ tool, PL-Marker,^105^ SciCo,^106^ and ScispaCy^107^ are frequently applied.

##### Vector representations, similarity search, and retrieval (E2/E3)

A range of embedding generation methods is used to represent text, structured data, molecular properties, and KG nodes. Text embeddings are commonly produced using models such as Text-Embedding-3 (OpenAI),^123^ BERT,^94^ SciBERT,^95^ SentenceBERT^96^ (all-MiniLM-L6-v2), BAAI/bge-large-en-v1.5,^97^ Google-GenerativeAIEmbeddings,^98^ and jina-embedding-v3.^99^ Molecular similarity is computed using measures such as the Tanimoto coefficient^102^ based on fingerprints and ChemBERTa^100^ cosine similarity from last hidden state embeddings. For efficient similarity search and retrieval, vector indices and nearest neighbor search libraries such as FAISS^101^ are widely used, supporting fast retrieval of semantically or chemically similar items.

##### Cheminformatics and molecular analysis (H2)

Several specialized tools, such as RDKit^124^ for molecular feature calculation, OmegaFold^125^ for protein folding prediction, and SynergyFinder 3.0^126^ for evaluating drug synergy and additivity from experimental data, are used.

#### Benchmark datasets and evaluation corpora

##### Drug discovery, bioactivity, and clinical

Several benchmark datasets are widely used in drug discovery and clinical research. DrugBank^22^ and KIBA^23^ provide molecular and bioactivity data for drug-target interaction prediction, while DAVIS^108^ offers drug and protein binding affinity data. Large-scale compound collections such as ZINC^109^ (over 230 million purchasable 3D compounds) and ChEMBL^110^ (bioactive compounds with properties) are commonly used for training, activity optimization, and modification tasks. Protein-ligand docking performance is assessed using CrossDocked2020,^111^ and clinical evaluation often relies on MIMIC-CDM,^112^ which contains 2,400 patients with four abdominal conditions including patient histories, physical exam notes, imaging reports, and laboratory results.

##### Materials science and chemistry

In materials science, datasets such as MP-20 (a subset of Materials Project^40^ with 45,231 inorganic crystalline materials), NOMAD^39^ (2.9 million materials), Matbench,^114^ and JARVIS-DFT^113^ (61,541 crystal structures with band-gap values) are commonly used for property prediction and generative modeling. OC20-S2EF 2M^115^ provides two million catalyst structures for training models such as CatGPT. Additional chemistry-focused benchmarks include TOMATO-Chem,^2^ for hypothesis rediscovery and ranking from annotated papers, and Superconductor Optimization (SPO),^17^ which contains 26,321 superconducting materials with chemical formulas and critical temperatures.

##### Auxiliary multi-domain testbeds

Various multi-domain datasets are used for evaluation in natural language processing (NLP), AI reasoning, and behavioral tasks. BUSINESS SHOES^116^ evaluates binary classification for shoe suitability, and DECEPTIVE REVIEWS^117^ contains genuine and fake hotel reviews for deception detection. HEADLINE POPULARITY^118^ and TWEET POPULARITY^119^ assess popularity prediction of headlines and tweets, respectively, while DREADDIT^120^ and PERSUASIVE PAIRS^121^ focus on mental stress detection and persuasive argument prediction. Systematic review and reasoning datasets include RCT Summaries,^122^ which links randomized controlled trials to PubMed papers, and HypoGen,^45^ containing approximately 5,500 structured problem-hypothesis pairs from NeurIPS 2023 and ICLR 2024 papers, used for fine-tuning and evaluation.

#### How these resources connect to EXHYTE

E2 (data retrieval) is powered by literature and dataset retrieval APIs (Semantic Scholar,^81^ PubMed,^82^ arXiv,^83^ OpenAlex,^84^ and PubChem^85^) and efficient similarity search tools such as FIASS,^101^ Tanimoto coefficient,^102^ etc. E3 (knowledge assembly) leverages PyPDF2,^87^ SciPDF^88^ for parsing documents, RecursiveCharacterTextSplitter^91^ for document chunking, pymupdf4llm,^89^ S2ORC-doc2json^90^ for documents formatting, BLINK,^103^ NER,^104^ PL-Marker,^105^ and SciCo^106^ for entity extraction and normalization. H2 (prioritization) draws on similarity metrics (Tanimoto^102^ and ChemBERTa^100^) and benchmark corpora (e.g., TOMATO-Chem^2^ and Matbench^114^) to score plausibility and novelty against known neighborhoods. T1-T2 (test and refinement) use domain datasets (OC20-S2EF,^115^ JARVIS-DFT,^113^ MIMIC-CDM,^112^ and ZINC^109^) to instantiate executable protocols, measure performance with domain metrics, and iterate using empirical feedback.

A typical *in silico* screening loop begins by validating textual inputs (molecular SMILE string using RDKit^124^), and PyPDF2,^87^ SciPDF,^88^ and pymupdf4llm^89^ are used for related PDF documents parsing and content extraction supported by structured conversion pipelines such as S2ORC-doc2json^90^ and the RecursiveCharacterTextSplitter.^91^ Candidate representations are then retrieved through embedding-based similarity search, using FAISS^101^ for vector indexing and retrieval, Tanimoto^102^ fingerprint similarity or ChemBERTa^100^ latent-space cosine similarity for molecular comparison, and text embeddings generated via models such as Text-Embedding-3,^98^ SciBERT,^95^ or BAAI/bge-large-en-v1.5.^97^ Task-relevant features are extracted and normalized via entity recognition and linking tools including BLINK,^103^ NER,^104^ PL-Marker,^105^ SciCo,^106^ and ScispaCy.^107^ Candidate hypotheses or molecular designs are subsequently evaluated on domain-specific benchmark datasets—such as Matbench^114^ or MP-20^40^ providing quantitative feedback for H2 prioritization and informing iterative refinement under T1-T2. This loop enables EXHYTE systems to continuously integrate retrieval (E2), structured knowledge assembly (E3), and hypothesis ranking (H2) within a unified testing and improvement cycle.

Together, these tools and datasets provide the material on which the EXHYTE strategies operate, enabling systems to move from retrieval and assembly to principled prioritization, testing, and refinement across chemistry, materials, biomedicine, and general scientific reasoning.

## Discussion and future outlook

This survey synthesizes recent work on AI for scientific discovery through the EXHYTE framework, an operational workflow lens for organizing how LLMs and agentic systems support (or approximate) *Explore*, *Hypothesize*, and *Test* functions. Using this lens, we unify a fast-growing and heterogeneous literature, surface stage-specific strengths and gaps, and highlight where current systems fail to compose into robust end-to-end discovery workflows. Because EXHYTE organizes systems by workflow function rather than by agent architecture, the same lens applies to role-partitioned multi-agent scaffolds and to the newer paradigms now entering the field, tool-use systems, standardized tool-access (MCP-style) protocols, coding agents with explicit plan-then-execute loops, and computer-use agents that drive software and instruments through their interfaces. Rather than requiring a new stage for each architecture, these map onto existing substages: tool use corresponds to retrieval and knowledge assembly (E2-E3) and tool-grounded experimental design (T1), plan-then-execute behavior to protocol generation and computational testing (T1–T2), and reflection or critique mechanisms to prioritization and refinement (H2, T2).

### Stage-level maturity and bottlenecks

Our synthesis finds that Explore is the most mature stage, driven by rapid progress in literature retrieval, RAG pipelines, knowledge-graph construction, and representation learning. However, this maturity is uneven: most systems treat literature as the primary evidence source, while comparatively few analyze real scientific datasets to identify patterns, anomalies, confounders, or mechanistic gaps that would initiate genuinely data-driven discovery. In contrast, Hypothesize remains underdeveloped beyond generation: many systems can propose ideas, but fewer provide rigorous mechanisms for novelty checking, plausibility assessment, or principled prioritization under explicit constraints. The weakest link remains Hypothesize → Test: translating promising hypotheses into executable experimental plans with reliable evaluation signals and then using those signals to refine or reject hypotheses. The Test stage also lags in practice: most systems remain *in silico*, and the few that incorporate wet-lab validation typically depend on substantial human supervision and bespoke infrastructure. Two further gaps sit at the workflow’s edges rather than within any single stage: trigger formation, problem finding, and assessment of which questions are worth pursuing remain largely human-supplied, since systems enter through human-defined inputs rather than identifying their own problems; and even systems that connect multiple stages stop short of full automation, because evidence interpretation, hypothesis revision, and scientific value judgment still require human checkpoints.

### Scope and limitations of EXHYTE as an operational lens

EXHYTE is not intended as a comprehensive theory of all scientific change. It best characterizes data-intensive, tool-mediated inquiry where progress proceeds through iterative exploration, hypothesis formulation, and empirical appraisal. Important dimensions of scientific practice are only partially represented in current systems mapped onto EXHYTE. First, paradigm shifts and other forms of “revolutionary” change involve broader conceptual re-framings and shifts in standards of evidence that are not well captured by present-day computational workflows. Second, scientific knowledge is stabilized through external validation and organized skepticism, including community scrutiny, replication, and credit/prioritization mechanisms, which extend beyond closed-loop internal scoring. Third, human scientific judgment extends well beyond ranking or approving machine-generated outputs: domain intuition, problem selection, interpretive judgment, and contextual understanding shape what counts as a meaningful research question and what constitutes adequate evidence. This last point also carries a risk that grows as workflows automate more substages: heavy reliance on AI for retrieval, idea generation, and appraisal may erode the very capacities—critical reading, independent judgment, and the ability to frame and recognize a worthwhile problem—that these systems presuppose in their human users. We therefore treat the preservation of human judgment not as a temporary scaffold to be removed as systems mature but as a standing requirement for trustworthy discovery. Throughout this survey, we accordingly treat EXHYTE as a pragmatic framework for mapping AI system capabilities and integration points, while explicitly noting where essential social and epistemic mechanisms remain outside the loop. In addition, ethical and governance considerations (safety, dual-use risk, provenance/consent, and accountability) impose cross-cutting constraints on what should be attempted, what can be trusted, and how EXHYTE workflows should be audited in practice.

### Robustness, evaluation, and failure modes

A critical challenge across the literature is that evaluation often relies on proxies (e.g., novelty scores, benchmark performance, and simulated rewards) that may not track scientific value, causal adequacy, or real-world reproducibility. Generative components introduce risks of hallucination, error propagation, and “plausible but wrong” mechanistic narratives that can be amplified by retrieval and tool use. A related and more subtle risk is epistemic rather than factual: because these systems are grounded in published literature, available datasets, and user-specified goals, they tend to inherit the scope and assumptions of those inputs. This can narrow hypothesis generation toward well-represented, LLM-familiar regions of the literature and reinforce dominant paradigms, while underweighting minority findings, negative results, or theory-laden framings that fall outside the training and retrieval distribution. Left unexamined, such systems may appear productive while systematically contracting the space of hypotheses a researcher considers. Many systems remain proof-of-concept demonstrations, and few have been tested for robustness across domains, sensitivity to data quality, or stability under distribution shift. Reproducibility is also uneven: end-to-end pipelines can be difficult to re-run due to changing APIs, non-deterministic generation, incomplete reporting of prompts/tools, and hidden pre-processing steps. These issues motivate evaluation protocols that go beyond task completion and linguistic or internal-consistency metrics to also assess traceability (evidence linking), uncertainty calibration, reproducible execution, and outcome-based validation, together with the breadth of the hypothesis space a system explores, its robustness to biased or theory-laden inputs, and the degree to which it preserves transparency and meaningful human oversight. By these criteria, a system that completes more workflow stages is not necessarily more trustworthy than one that completes fewer while keeping its reasoning legible and its hypothesis space open.

### Outlook: Actionable directions from EXHYTE bottlenecks

The clearest trajectory for closing the H → T (hypothesis-to-test) gap is the move toward closed-loop, self-driving laboratories, in which hypothesis generation, experiment design, execution, and interpretation are chained into a continuous active-learning cycle. Several systems in our corpus already take partial steps in this direction: Chemist-X^1^ and ChatBattery^78^ execute and characterize candidates through automated or wet-lab experiments, Perovskite-R1^46^ fabricates and evaluates complete devices, Robin^26^ operates a lab-in-the-loop with human-executed assays, and Brunnsåker et al.^79^ run auto-formalized protocols on an automated cell-culture and mass spectrometry platform. Emerging systems beyond our corpus point to a complementary route: LabOS,^127^ for example, couples a self-evolving multi-agent digital core with an extended-reality interface through which the AI perceives the physical bench and guides a human experimenter in real time, closing the loop through human-AI perceptual collaboration rather than full robotic automation. Relative to these platforms, most systems analyzed through EXHYTE remain *in silico* and stop short of autonomous experiment selection and execution. This contrast sharpens our diagnosis of the bottleneck: progress will depend less on additional generative capacity than on standardized experiment interfaces, active-learning-driven choice of which experiment to run next, and reliable automated interpretation of noisy empirical results, capabilities that self-driving labs operationalize but that remain only partially present in current AI-for-science systems.

This diagnosis points to a focused research agenda organized around the EXHYTE transitions. First, E → H (evidence-to-hypothesis) requires methods that move beyond literature summarization toward data-grounded anomaly detection, causal abstraction, and explicit representation of uncertainty and confounding. Second, H → T requires standardized experiment interfaces and planners that translate hypotheses into executable protocols with explicit assumptions, constraints, and measurable endpoints; that select which experiment to run next under resource and uncertainty constraints through active-learning or experimental-design strategies; and that apply automated checks for feasibility, safety, and reproducibility. Third, T → H/E (feedback-to-revision) requires reliable interpretation of noisy empirical results—experimental, computational, or statistical—that supports principled refinement, hypothesis pruning, and model updating rather than ad hoc regeneration. Finally, credible progress will require incorporating external validation mechanisms into AI workflows, including replication-oriented evaluation, transparent provenance of evidence, and community-facing artifacts that enable scrutiny and credit assignment. Taken together, these directions shift emphasis from isolated task automation toward integrative scientific intelligence: systems that can ground hypotheses in evidence, execute and document tests reproducibly, and revise beliefs in response to reliable feedback, while remaining legible and controllable to human scientists.

### Interactive website

A companion website is available at https://webapps.crc.pitt.edu/exhyte/, providing description of the EXHYTE framework and summaries of all collected papers and offering interactive tools that allow users to generate customized literature surveys based on their selected papers. The paper list will be continuously updated to reflect new developments in the field.

## Acknowledgments

This research was supported by grants from the 10.13039/100000002National Institutes of Health (U01CA279618 and R21GM155774 to Y.H.; CA096512, CA284554, CA278812, CA291244, and CA124332 to S.-J.G.; and R35GM154967 and R03CA305794 to Y.-C.C.), 10.13039/100030715UPMC Hillman Cancer Center Startup Funds to S.-J.G. and Y.H., and in part by award P30CA047904. This research was also supported in part by the 10.13039/100016300University of Pittsburgh Center for Research Computing, RRID: SCR022735. Specifically, this work used the HTC cluster, which is supported by S10OD028483.

## Author contributions

M.M.H. and Y.H. conceptualized the paper and prepared the initial draft. S.J. contributed to the figures, J.S. prepared the tables, and H.H. prepared the website. All authors participated in paper survey and discussions, edited the paper, and approved the final version of the paper.

## Declaration of interests

The authors declare no competing interests.

## Declaration of generative AI and AI-assisted technologies in the writing process

During the preparation of this work, the authors used OpenAI GPT-4.1 with customized prompts to generate initial summaries of the papers reviewed in this survey. All AI-generated summaries were subsequently reviewed and edited by the authors. The authors take full responsibility for the accuracy, interpretation, and final content of the publication.

## Supplemental information

Table S1. Summary of reviewed papers with check marks which strategies were employed by each paper
